# Ureteric obstruction secondary to schistosomiasis 2 years after praziquantel therapy: a case report

**Published:** 2012-06-17

**Authors:** Abimbola Olaniyi Olajide, Folakemi Olajumoke Olajide, Ademola Adegoke Aremu, Akinkunmi Oluwole Komolafe

**Affiliations:** 1 Department of Surgery, Ladoke Akintola University of Technology, Ogbomoso, Nigeria; 2 Department of Community Health, Obafemi Awolowo University, Ile-Ife, Nigeria; 3 Department of Radiology, Ladoke Akintola University of Technology, Ogbomoso, Nigeria; 4 Department of Morbid Anatomy, Obafemi Awolowo University, Ile-Ife, Nigeria

**Keywords:** Urethra, obstruction, schistosomiasis, praziquantel, surgery

## Abstract

Schistosomiasis is one of the oldest and commonest parasitic infestations of mankind and one of the leading infestations of public health concern. Hitherto, praziquantel has been the only drug for mass eradication of Schistosomiasis. Few reports have shown possible resistance of the schistosome to praziquantel in some part of the world with global concern about the future of the drug. We report this case to illustrate progression of Schistosomiasis to surgical complication despite treatment, with bizarre presentation misleading attending physicians. Diagnosis was finally confirmed at post-operation histological examination of specimen.

## Introduction

Schistosomiasis is one of the oldest diseases known to mankind and the commonest tropical parasitic infection affecting the urogenital system. Despite its widespread distribution; symptomatic infection is found in only few infected people while majority are asymptomatic. This is responsible for the low mortality rate and the often underestimated health significance of the parasitic infection [1]. *Schistosoma haematobium* characteristically affects the urinary tract with painless terminal haematuria as the commonest mode of presentation and the main target organs being the bladder and distal ureters. Praziquantel has remained the only drug used in mass control of schistosomiasis because it is cheap and effective. However, reports on resistance to this drug in some parts of the world have been a source of public health concern [2]. We report this case to illustrate progression of pathology in schistosomiasis despite praziquantel therapy and to alert surgeon in endemic regions of the need to increase their index of suspicion to prevent misdiagnosis.

## Patient and case report

A 23- year old male Nigerian undergraduate presented with 12 months history of colicky left loin pain of insidious onset which has slowly but progressively worsened. There was no lower urinary tract symptom, haematuria, loin swelling, weight loss, cough and no previous retroperitoneal surgery. He grew up in a rural community of south western Nigeria where he swam in local shallow stream which is the main source of water in the community. Two years before presentation, he had painless, terminal haematuria which was treated with praziquantel with complete clinical resolution of haematuria. Based on the medical counselling given, he never swam in shallow stream after the treatment and has since relocated from the rural area to an urban area. Renal ultrasonography on presentation showed dilatation of the renal calyces and pelvis on the left side with grossly normal right kidney and ureter. Urine microscopy showed no microscopic haematuria and 4–5 white blood cells per high power field. There were no ova or parasite seen in the urine and no bacterial growth on incubation. Serum electrolyte, urea and creatinine were all within normal limits.

**Figure 1:**
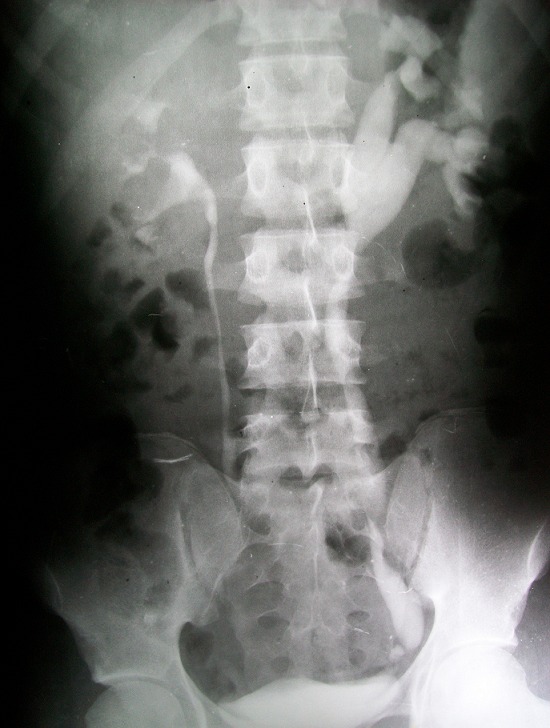
Intravenous urography showing dilated left pelvi-calyceal system and ureter with areas of stenosis at the pelvic portion of the ureter

**Figure 2:**
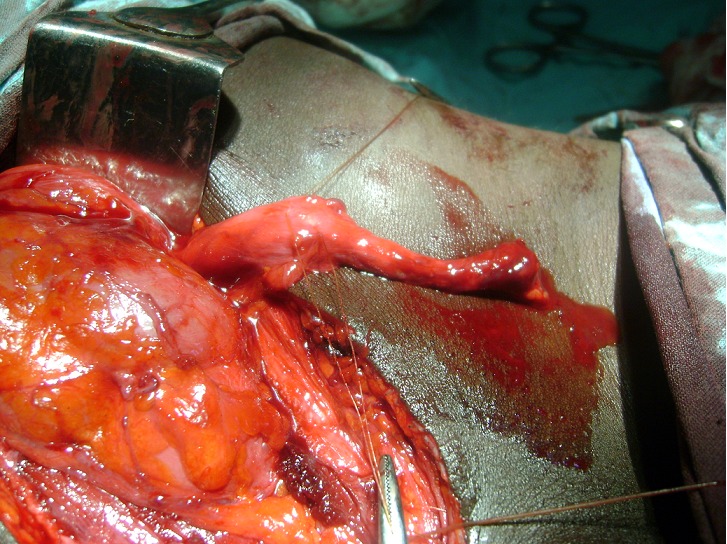
Intraoperative picture showing the obstructed distal segment of the ureter in contrast with the dilated proximal segment

Intravenous urography (Figure 1) showed dilatation of the left renal calyces, pelvis and ureter. There were 2 areas of stenosis at the distal portion of the left ureter (Figure 1). The plane film also showed calcification at the area of obstruction. Initial diagnosis of ureteric obstruction secondary to calculi was made. Intra-operatively, the findings on urography were confirmed, no calculus was seen and the bladder was grossly normal (Figure 2). Resection of the strictured segment with left ureteroneocystostomy and psoas hitch was done. Post operative period was uneventful and patient was discharged from the hospital on the 8th post operative day.

**Figure 3:**
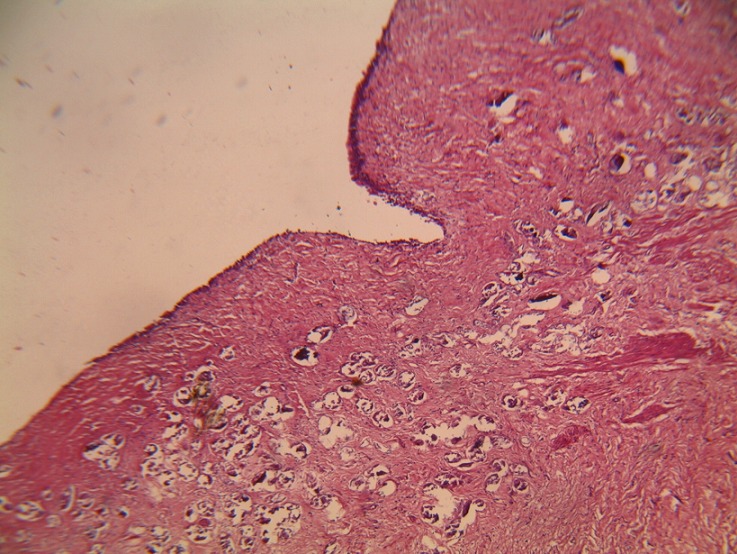
Photomicrograph of the resected segment showing multiple ova of schistosomiasis in the wall of the ureter

Histopathology report however showed a left ureteric mass with numerous Schistosoma ova within the ureteric wall (Figure 3). He was subsequently treated with a repeat dose of praziquantel and has been on follow up for over 3 years with no related complaints.

## Discussion

Despite major efforts in control, schistosomiasis continues to be one of the most prevalent parasitic infections in the world, especially in sub-Saharan Africa. The clinical manifestations result from host response to the ova which get deposited in tissues with fibrosis, hyalinization, calcification and subsequent destruction of the ova with granuloma formation. Myriads of presentation of ureteric lesions in schistosomiasis can mislead the attending physician if there is no high index of suspicion. Multiple areas of stenosis (beaded appearance) could suggest tuberculosis of the upper urinary tract and calcification of lesion could suggest calculi [3].

Praziquantel, though the only effective drug currently used in mass eradication of schistosomiasis, has been reported to yield a lower cure rates than expected in some parts of Africa [4]. There are 2 reports of incomplete clearance of schistosomiasis acquired by travellers within an endemic setting, who used appropriate doses of praziquantel upon return to nonendemic areas [5, 6]. Though the reports are still limited to some parts of Africa, resistance to praziquantel is becoming a major public health concern and the future of praziquantel in treatment and eradication of scistosomiasis is doubtful [4]. Various reasons have been suggested for the resistance: emergence of resistant strains, presence of immature flukes at the time of treatment or re-infection of the patients [3]. Resolution of Haematuria and absence of ova of the parasite in urine could have misled the attending physician to believe there was complete clearance of the ova with the initial treatment while the pathology progresses to cause ureteric obstruction. Urine examination is simple, safe and cheap but it can also give a false negative impression because the ova may be absent in urine in chronic cases. This has made some researchers to suggest eosinophiluria as an index to diagnose urinary schistosomiasis, which could be of help in diagnosis of such cases when suspected [7]. There have been debates on the future of praziquantel in control of schistosomiasis because of anticipation of resistance which is being increasingly reported from endemic areas especially in Africa [8]. As the only effective routinely used drug for treatment and control of this disease, development of resistance is a great threat to the battle against the menace of schistosomiasis. Praziquantel has been shown to be relatively ineffective against juvenile schistosomes which may contribute to the poor cure rate and treatment failure in areas with high rates of transmission like endemic areas of Africa. This brought about the suggestion and adoption of two courses of praziquantel separated by a short interval of 2–4 weeks in some areas with resultant higher cumulative cure rates [9]. There is therefore need for closer monitoring of the use of praziquantel and a desperate need for development of some alternative compounds if control of this disease is to be achieved and sustained [10].

## Conclusion

Despite the effectiveness of praziquantel in treatment of schistosomiasis, resistant schistosomes could have evolved with disease progression despite therapy. Resolution of haematuria and absence of ova in urine may not suggest complete resolution of infection. Thus, awareness of these possibilities and high index of suspicion is therefore necessary to prevent disease progression and serious complications.
